# Development of a Novel Immune Infiltration-Related ceRNA Network and Prognostic Model for Sarcoma

**DOI:** 10.3389/fcell.2021.652300

**Published:** 2021-07-01

**Authors:** Deyao Shi, Shidai Mu, Feifei Pu, Binlong Zhong, Binwu Hu, Jianxiang Liu, Tongchuan He, Zhicai Zhang, Zengwu Shao

**Affiliations:** ^1^Department of Orthopaedics, Union Hospital, Tongji Medical College, Huazhong University of Science and Technology, Wuhan, China; ^2^Molecular Oncology Laboratory, Department of Orthopaedic Surgery and Rehabilitation Medicine, The University of Chicago Medical Center, Chicago, IL, United States; ^3^Institute of Hematology, Union Hospital, Tongji Medical College, Huazhong University of Science and Technology, Wuhan, China

**Keywords:** sarcoma, prognostic risk model, ceRNA network, tumor microenvironment, nomogram

## Abstract

Due to the rarity and heterogeneity, it is challenging to explore and develop new therapeutic targets for patients with sarcoma. Recently, immune cell infiltration in the tumor microenvironment (TME) was widely studied, which provided a novel potential approach for cancer treatment. The competing endogenous RNA (ceRNA) regulatory network has been reported as a critical molecular mechanism of tumor development. However, the role of the ceRNA regulatory network in the TME of sarcoma remains unclear. In this study, gene expression data and clinical information were obtained from The Cancer Genome Atlas (TCGA) sarcoma datasets, and an immune infiltration-related ceRNA network was constructed, which comprised 14 lncRNAs, 13 miRNAs, and 23 mRNAs. Afterward, we constructed an immune infiltration-related risk score model based on the expression of IRF1, MFNG, hsa-miR-940, and hsa-miR-378a-5p, presenting a promising performance in predicting the prognosis of patients with sarcoma.

## Introduction

Sarcomas are heterogeneous malignancies of mesenchymal origin, accounting for 1% of adult cancers, which are classified into more than 175 distinct subtypes (Steele and Pillay, 2020). It is difficult to make impressive progression in new therapeutic approaches for patients with sarcoma because of the rarity and heterogeneity (Miwa et al., 2019; Gamboa et al., 2020; Grünewald et al., 2020). Although the combination of resection surgery and multidrug adjuvant chemotherapy has improved the 5-year survival probability of soft tissue sarcoma to 60–80%, about 25% of patients develop metastatic disease after curative treatment for the primary tumor, and approximately 10% of patients are found with metastatic lesions at the time of diagnosis (Gamboa et al., 2020; Hashimoto et al., 2020; Heng et al., 2020). Therefore, it is of pivotal significance to explore potential molecular mechanisms and identify critical therapeutic targets in sarcoma.

The tumor microenvironment (TME), comprising extracellular matrix (ECM) and cellular components, has been documented to be firmly associated with the initiation and progression of sarcoma (Heymann et al., 2019). Combined regimens based on immune checkpoint inhibitors (anti-PD1 or anti-CTLA4) and modified T-cell therapies are currently being tested in specific sarcoma subtypes with a significant clinical benefit for the patients (Pollack et al., 2018; Dyson et al., 2019; Heymann et al., 2020). However, the immune microenvironment in sarcoma substantially differs from other immune-responsive tumors such as melanoma. Based on a transcriptomic analysis of the cell population in TME, sarcoma can be classified into five different classes, sarcoma immune classes (*SIC*) from A (immune desert), C (vasculature), to E (immune and tertiary lymphoid structures), where patients with *SIC* A showed worse overall survival (OS) than *SIC* E (*P* = 0.025) (Becht et al., 2016). Recently, a series of novel algorithms such as ESTIMATE (Yoshihara et al., 2013) and Cibersort (Chen et al., 2018) have been publicly released to analyze the infiltrating stromal and immune cells in TME based on gene expression data, which helps to study the functioning roles of TME in tumor initiation and progression (Yoshihara et al., 2013).

Accumulating evidence has shown that transcriptional regulation between mRNAs and ncRNAs plays a crucial role in sarcoma progression, including proliferation, migration, metastasis, and multidrug resistance (Wang et al., 2018, 2019; Xie et al., 2018; Ma et al., 2019). Competing endogenous RNA (ceRNA) networks have been reported as an important mechanism to explain posttranscriptional regulation. Zhu et al. (2019) constructed ceRNA regulatory networks of both lncRNA–miRNA–mRNA and circRNA–miRNA–mRNA interactions to investigate the underlying mechanisms of chemoresistance in osteosarcoma. Zhang et al. (2019) identified three lncRNAs and two miRNAs regulating three mRNAs in a ceRNA network as promising prognostic biomarkers of osteosarcoma recurrence. The research on ceRNA networks in sarcoma was generally based on the differential genes screening between tumor and normal controls, but there were few studies reporting ceRNA networks related to TME of sarcoma.

Weighted gene co-expression network analysis (WGCNA) (Langfelder and Horvath, 2008) is a practical algorithm identifying highly related genes and aggregating them into the same genetic module, which is commonly used to investigate the correlation between gene sets and clinical characteristics, thus identifying potential biomarker candidates or new therapeutic targets from genetic data (Maertens et al., 2018). In this study, we calculated the infiltrating immune and stromal scores of sarcoma cases in The Cancer Genome Atlas (TCGA) (Grossman et al., 2016) using the ESTIMATE algorithm, identified the modules most relevant to the TME of sarcoma through WGCNA, and then established an immune infiltration lncRNA–miRNA–mRNA ceRNA network to screen genes of clinical significance. Furthermore, we constructed a prognostic risk score model and a nomogram based on the expression of immune infiltration-related genes. These findings will provide new insights for the regulatory mechanisms of the tumor immune microenvironment in sarcoma progression, as well as identify promising clues in developing the TME related therapeutic targets for patients with sarcoma.

## Results

### Association of Immune Infiltration and Clinical Outcomes of Sarcoma

**FIGURE 1:**
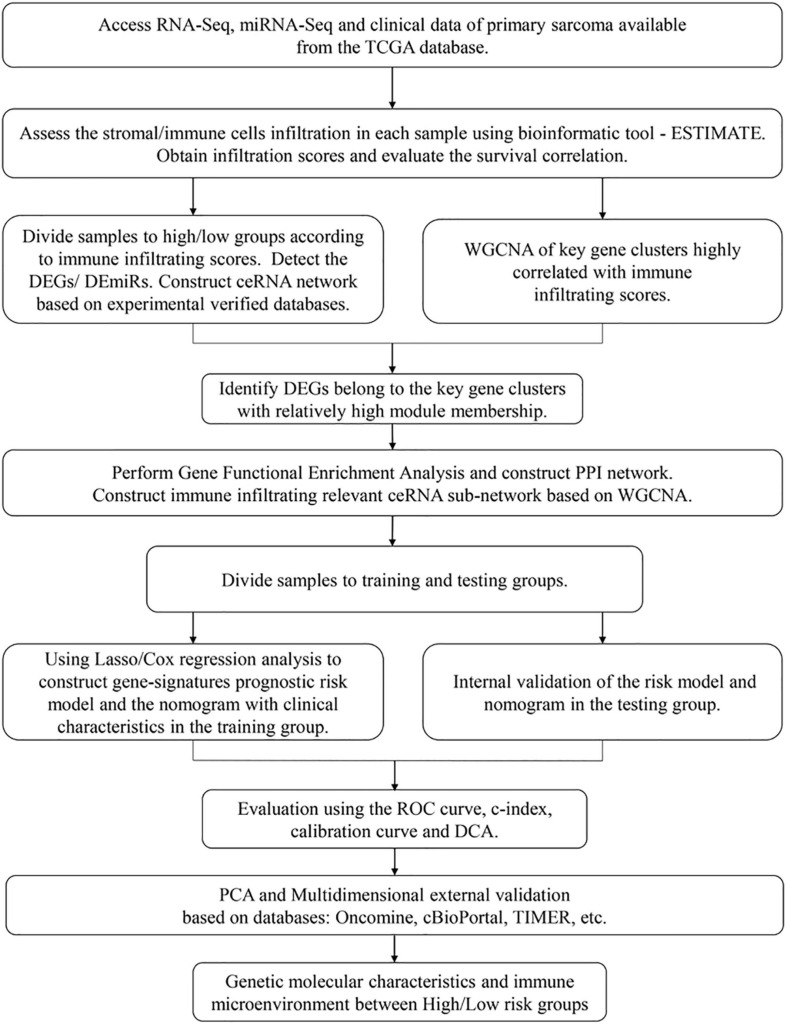
A flowchart for the process of the present study.

A flowchart was diagramed to demonstrate the procedure of our study (Figure 1). RNA-seq data and matched clinical information of 259 sarcoma patients were obtained from the TCGA database. The ESTIMATE algorithm was utilized to evaluate tumor purity and immune/stromal cell infiltration in the samples by calculating corresponding scores (the ESTIMATE score indicated tumor purity and the immune/stromal scores indicated immune/stromal cells infiltration). The ESTIMATE scores ranged from –3,088.65 to 5,077.57 (median = 1,320.73), the immune scores ranged from –1,953.32 to 3,212.09 (median = 339.05), and the stromal scores ranged from –1,214.15 to 2,460.46 (median = 988.55). Among the sarcoma patients, 118 (45.56%) were male and 141 (54.44%) were female. The age of patients at initial diagnosis ranged from 27 to 90 (median = 61). In the aspect of survival status, 161 (62.16%) patients were alive and 98 (37.84%) patients were dead. Other clinical characteristics including race, follow-up period, histological type, tumor margin status, tumor depth, local disease recurrence, metastasis at diagnosis, radiation therapy, and tumor necrosis percentages were all documented (Supplementary Table 1).

**FIGURE 2:**
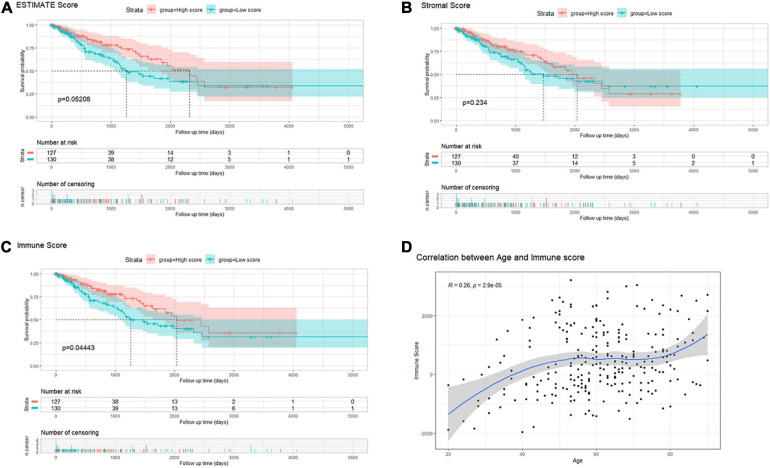
**(A–C)** Kaplan–Meier survival analysis of sarcoma patients’ overall survival according to scores calculated by the ESTIMATE algorithm: ESTIMATE (tumor purity), stromal cell infiltration, and immune cell infiltration. **(D)** The correlation between immune scores and the age of diagnosis in patients with sarcoma.

By setting the median as cutoff values, 259 sarcoma samples were divided into low/high ESTIMATE score groups, low/high Stromal score groups, and low/high Immune score groups. Survival analysis indicated that there was no significant difference between low/high ESTIMATE score and low/high Stromal score groups (log-rank *P* = 0.05206 and 0.234; Figures 2A,B). However, OS probability was significantly higher in the high immune score group (log-rank *P* = 0.04443; Figure 2C). We further investigated the association between immune score and clinical characteristics. We found that age of initial diagnosis was positively correlated with immune score (*R* = 0.26, *P* = 2.9e-05; Figure 2D). Other clinical characteristics including tumor margin status, tumor depth, local disease recurrence, metastasis at diagnosis, radiation therapy, and tumor necrosis percentages were not significantly associated with immune score (Supplementary Figure 1).

### Identification of Differentially Expressed Genes

**FIGURE 3:**
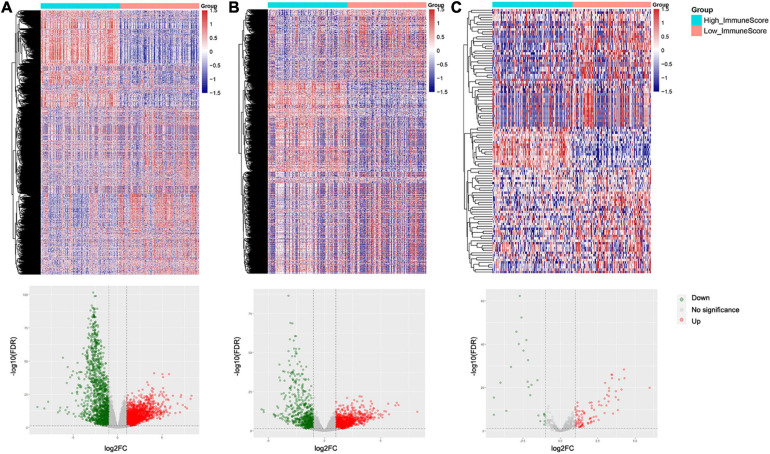
Heatmaps and volcano plots of differentially expressed genes and miRNAs between the high and low immune score groups: **(A)** mRNAs, **(B)** lncRNAs, and **(C)** miRNAs.

Differentially expressed genes (DEGs) between high and low immune score groups were analyzed following the criteria of |log_2_FC| > 1 and FDR value < 0.05. A total 6,701 genes (4,000 upregulated and 2,701 downregulated) were detected significantly differentially expressed in the RNA-seq data (Supplementary Figure 2), among which 3,535 were mRNAs (2,063 upregulated and 1,472 downregulated) and 1,854 were lncRNAs (1,138 upregulated and 716 downregulated) (Figures 3A,B). Besides, 110 miRNAs (86 upregulated and 24 downregulated) were detected significantly differentially expressed in the miRNA-seq data (Figure 3C). Details were documented in Supplementary Table 2.

### WGCNA and Identification of the Immune Infiltration-Related Gene Module

**FIGURE 4:**
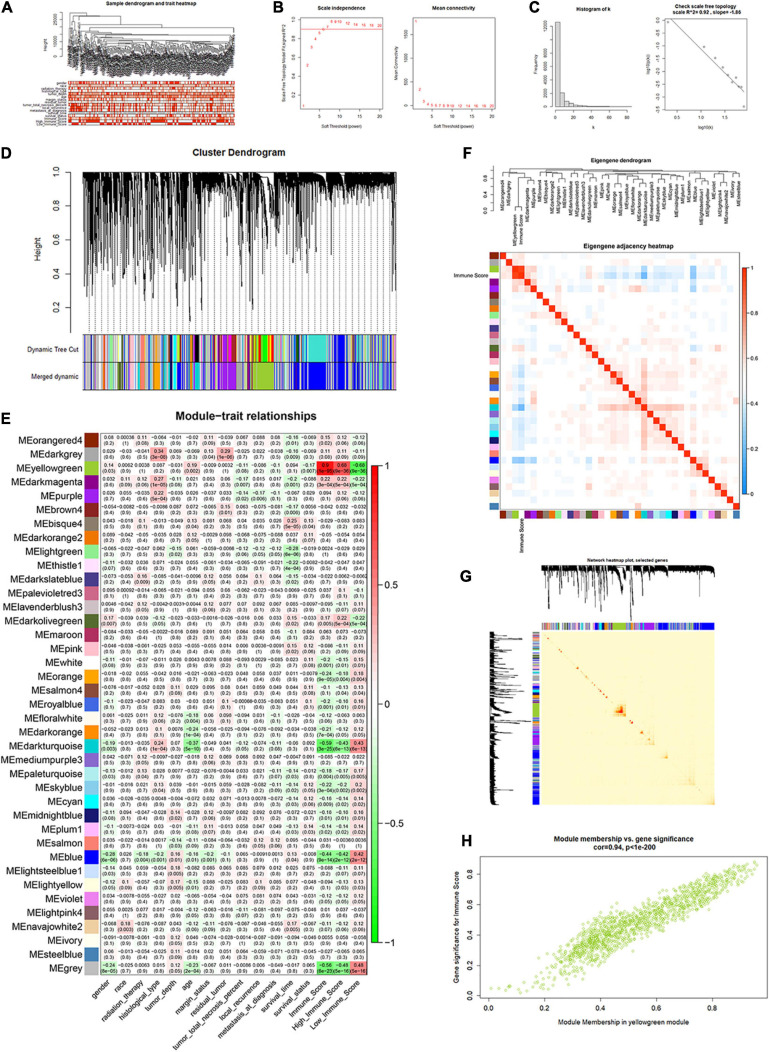
Identification of immune score-related gene clusters by WGCNA. **(A)** Sample dendrogram and trait heatmap. **(B,C)** Calculation and evaluation of the best β value in the co-expression network. **(D)** Merged dynamic gene cluster dendrogram. **(E)** Analysis and visualization of the module–trait relationship. **(F)** Eigengene adjacency heatmap with immune score included. **(G)** The topological overlap measure (TOM) for gene network connections (randomly selected 1,000 genes). **(H)** Correlation between gene module membership and gene significance for immune score in the yellow-green module (correlation coefficient = 0.94, *P* < 0.0001).

All lncRNAs and mRNAs with the top 50% variance among samples were included in WGCNA, with one sample detected as the outlier in the sample clustering procedure. For the retained 258 samples, all clinical characteristics and immune score were included as trait variables (Figure 4A and Supplementary Figure 3). The best β value in the co-expression network was calculated to be 7. Next, the method of dynamic tree cutting was used to further generate gene co-expression modules. The index for clustering of module eigengenes was modified to be 0.65 so that we can construct a reasonable number of merged modules (Figures 4B–D). As shown in the module–trait relationship figure, the eigengene adjacency heatmap, and the topological overlap measure (TOM) figure, the yellow-green module possessed the highest correlation with immune scores (*R* = 0.90, *P* < 0.0001) (Figures 4E–G). For the total 1,414 genes of this module, we observed a high correlation (*R* = 0.94, *P* < 0.0001) between gene significance of immune score and gene module membership (Figure 4H). Therefore, we identified the yellow-green module as the hub gene module related to immune infiltration. Moreover, we screened the top 30% genes (414 genes) in the yellow-green module as the hub gene sets for further study, by setting 0.65 of module membership as the threshold value (Supplementary Table 3).

### Construction of an Immune Infiltration-Related ceRNA Network

**FIGURE 5:**
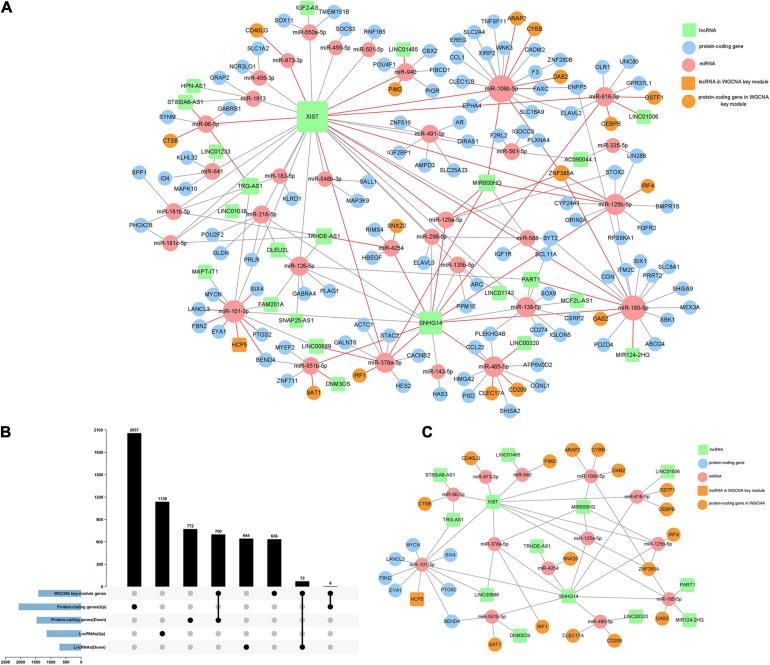
Construction of the immune infiltration-related ceRNA network. **(A)** A ceRNA network constructed by DEGs between high and low immune score groups (120 mRNA, 25 lncRNAs, and 33 miRNAs). **(B)** Intersections of DEGs and the key gene cluster (yellow-green module) identified by WGCNA. **(C)** A highly immune-infiltrating-related ceRNA subnetwork constructed by 23 mRNAs, 14 lncRNAs, and 13 miRNAs.

Based on the differentially expressed 1,854 lncRNAs, 110 miRNAs, and 3,535 mRNAs between high/low immune score groups, we constructed a ceRNA network by querying the RNA interaction relationship from databases using algorithm prediction (microT-CDS, miRDB) and experimental validated data (miRTarBase and lncbase v2). A total of 84 lncRNA–miRNA and 132 mRNA–miRNA interactions were identified, which comprised 25 lncRNAs, 33 common miRNAs, and 120 mRNAs. Besides, 778 DEGs were found in the yellow-green module of WGCNA by intersection. Genes belonging to the yellow-green module were highlighted in the ceRNA network (Figures 5A,B). By selecting these genes, an immune infiltration-related ceRNA subnetwork was constructed, which contained 14 lncRNAs, 13 miRNAs, and 23 mRNAs (Figure 5C).

### Gene Functional Enrichment Analysis and PPI Network Construction

**FIGURE 6:**
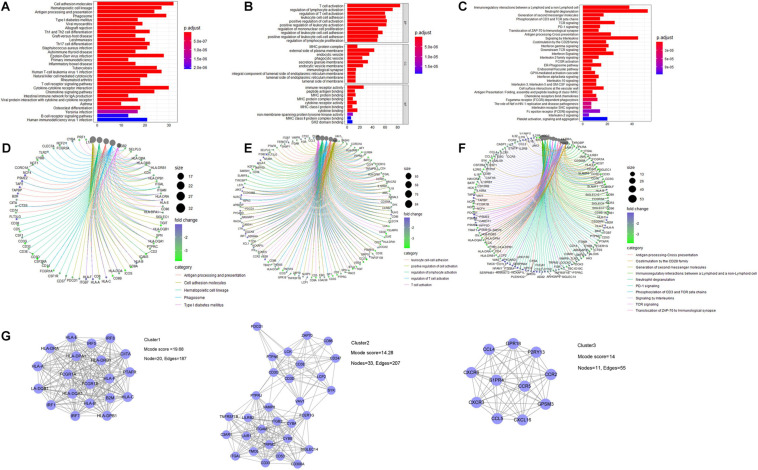
Gene functional enrichment analysis of identified genes related to immune infiltration. For KEGG pathway analysis: **(A)** bar plot and **(B)** gene-concept network plot for the over-representative analysis **(C,D)** for GO analysis, and **(E,F)** for Reactome pathway analysis. **(G)** PPI analysis was constructed by utilizing the STRING database, and three core subnetworks were identified via MCODE plugin.

For the gene functional enrichment analysis, we enrolled 778 genes by intersecting DEGs and the gene in the WGCNA yellow-green module. In the KEGG over-representation analysis (ORA), the top enriched entries were mainly immune-related pathways including antigen processing and presentation, and Th1/Th2 cell differentiation. In the GO ORA, enriched biological processes primarily belonged to immune-related GO terms such as T cell activation and regulation of lymphocyte activation. In the Reactome ORA, enriched pathways were also mainly immune related, such as phosphorylation of CD3 and TCR zeta chains. Besides, as shown in the gene-concept network, genes in the top enriched KEGG, Reactome pathways, and GO biological processes were mainly immune-related biomarkers (Figures 6A–F and Supplementary Table 4).

A PPI network *via* the STRING database was built to investigate the protein–protein interactions, which further identified three core clusters *via* the MCODE plugin. The first core PPI cluster with an Mcode score of 19.68 is composed of immune response-related proteins, such as the interferon regulatory factor (IRF) family members, human leukocyte antigen (HLA) family members, and Fc gamma receptors. The second core PPI cluster with an Mcode score of 14.28 comprises proteins highly associated with T cell immune response, such as CD3D, PDCD1, CD247, ZAP70, and ITGAL. The third core PPI cluster with an Mcode score of 14 is mainly composed of CXC chemokine receptors, C–C motif chemokine ligands, and receptors, which function in regulating lymphocyte chemotaxis and chemokine-mediated signaling pathway (Figure 6G and Supplementary Figure 4).

### Construction of an Immune Infiltration-Related Risk Score Model

**FIGURE 7:**
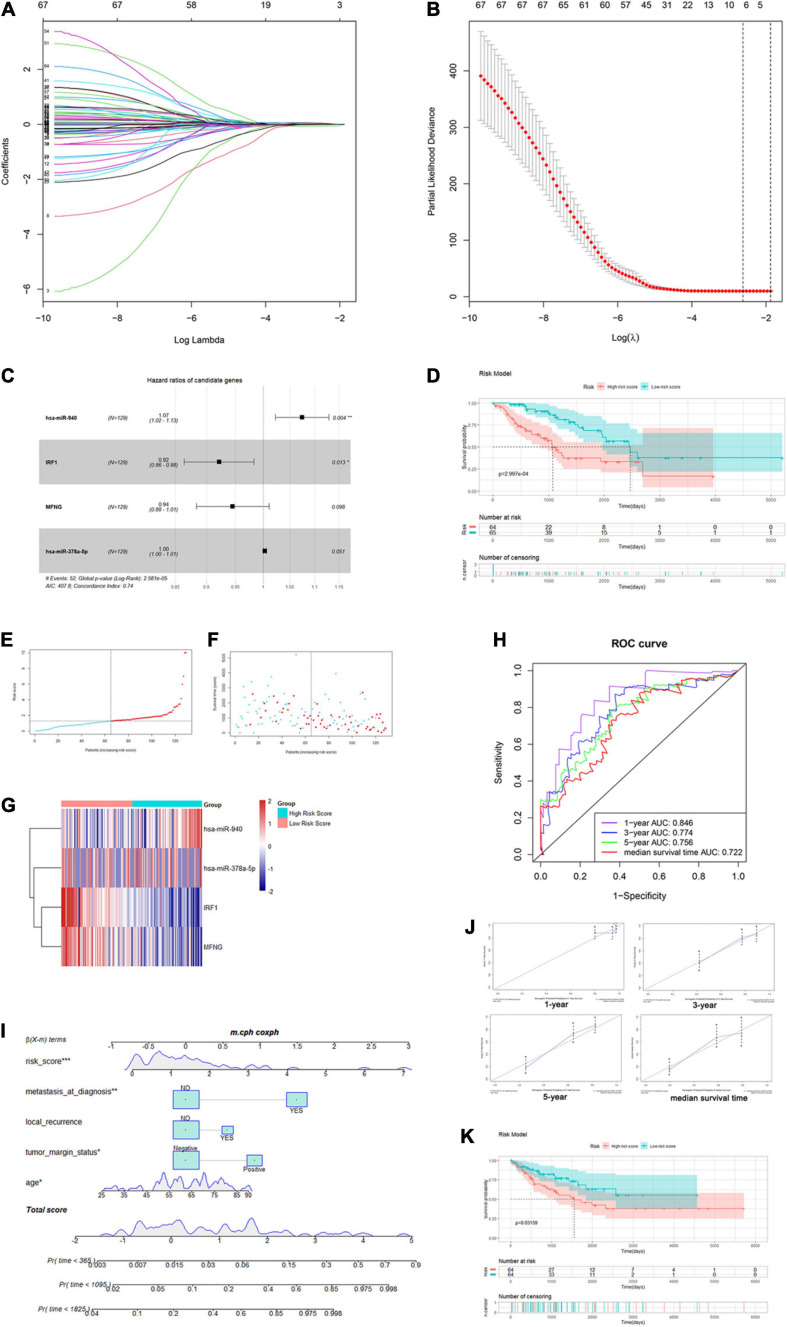
Construction of the immune infiltration-related risk score model and nomogram. **(A,B)** Plots for Lasso Cox regression analysis of genes identified by univariate Cox regression analysis. **(C)** Forest plot for the four genes in the most optimal model confirmed by multivariate Cox regression using the analytical method. **(D)** K–M survival analysis of training-cohort overall survival based on risk scores. **(E–G)** The risk score curve, survival status, and gene expression levels for each patient were discretely distributed in two groups. **(H)** Time-dependent ROC curves of the risk score model for predicting the survival probability of 1-, 3-, and 5-year and median-survival time overall survival. **(I)** Nomogram for predicting the survival probability of 1-, 3-, and 5-year overall survival. **(J)** The time-dependent calibration plots for the nomogram in the 1-, 3-, and 5-year time periods. **(K)** K–M survival analysis of testing-cohort overall survival according to risk score level.

We extracted the matched normalized RNA-seq data, normalized miRNA-seq data, and survival follow-up data of 257 sarcoma samples. Through the Caret R package, we randomly divided the TCGA sarcoma cases to training and testing cohorts. In the training cohort (*n* = 129), we integrated lncRNAs, miRNAs, and mRNAs in the immune infiltration-related ceRNA subnetwork and hub genes in the WGCNA yellow-green module to construct the immune infiltration risk score model. Univariate Cox regression analysis was firstly performed to identify 67 out of the 461 genes that were significantly associated with OS. Next, we applied the Lasso penalized Cox regression to construct a risk score model with optimal number of genes (Figures 7A,B). A total of five mRNAs and two miRNAs were identified and further analyzed with a stepwise multivariate Cox regression (Supplementary Table 5). The most optimal model with two mRNAs and two miRNAs was finally confirmed with the analytical method of AIC (Figure 7C). By summarizing the normalized expression of the two mRNAs and two miRNAs and the regression coefficient calculated from multivariate Cox regression analysis, a prognostic risk score model for prediction of OS was constructed using a formula as the following: risk score = (expression level of hsa-miR-940 ^∗^ 0.0719 + expression level of IRF1 ^∗^ (–0.0818) + expression level of MFNG ^∗^ (–0.0568) + expression level of hsa-miR-378a-5p ^∗^ 0.0028).

Then, we calculated the risk score for each patient and divided them into high- and low-risk groups using the median as the cutoff value. As shown in Figure 7D, Kaplan–Meier (K-M) survival analysis indicated that patients in the high-risk group had significantly shorter OS (log-rank *P* = 2.997e-04). The risk score, survival status, and gene expression levels were discretely distributed between two groups (Figures 7E–G). We further analyzed the AUC of time-dependent ROC curves. As shown in Figure 7H, AUCs of the risk score model were 0.846, 0.774, 0.756, and 0.722 for 1-, 3-, and 5-year and median survival time of all patients (5.455 years), respectively. In addition, the C-index of the risk score model was 0.74 (95% CI: 0.67–0.81, *P* = 8.02e-13). The results showed that the risk score model had a good capacity in OS prediction.

### Examination of the Risk Score Model as an Independent Prognostic Factor

In order to analyze the prognostic significance of the risk score model, we applied univariate and multivariate Cox regression analyses combining all available clinicopathologic factors in the training cohort, including age, gender, race, tumor depth, tumor margin status, tumor total necrosis percent, local recurrence, and metastasis at diagnosis. Univariate analysis identified that the risk scores and other five clinical characteristics were associated with OS with *P*-value < 0.1. Moreover, we enrolled these factors in the following multivariate analysis, which further identified that age (*P* = 0.004915), tumor margin status (*P* = 0.000628), local recurrence (*P* = 0.004733), metastasis at diagnosis (*P* = 0.002333), and risk score (*P* = 2.14e-05) were significantly associated with OS. Among these characteristics, risk score had the highest effect size (HR: 1.22, 95% CI: 1.11–1.33) (Supplementary Table 6). Thus, our result demonstrated that the immune infiltration-related risk score model was independent of conventional clinical characteristics.

### Construction and Evaluation of a Predictive Nomogram

Based on the five independent prognostics factors (risk score, age, tumor margin status, local recurrence, and metastasis at diagnosis), we developed a nomogram model to predict OS probability of sarcoma patients in 1-, 3-, and 5-year time periods. As shown in the nomogram plot in Figure 7I, risk score was presented as a major contributor compared to the other clinical characteristics. Time-dependent ROC analysis showed that AUCs of the nomogram were 0.658, 0.76, 0.786, and 0.747 for 1-, 3-, and 5-year and median survival time of all patients, respectively (Supplementary Figure 5G). The C-index for the nomogram was 0.74 (95% CI: 0.66–0.82, *P* = 3.92e-09). The time-dependent calibration plots showed that the bias-corrected lines for the nomogram were close to the standard line in 1-, 3-, and 5-year time periods (Figure 7J). These results indicated that the risk score model-based nomogram had an excellent capacity and consistency for OS prediction in the training cohort.

### Internal Validation of Immune Infiltration-Related Risk Score Model and Nomogram

The testing cohort (*n* = 128) was used for internal validation of the immune infiltration-related risk score model. The risk score for each patient was calculated using the same formula, and all patients were divided into high- and low-risk score groups likewise. The K–M survival curve showed that patients in the high-risk group also have significantly shorter OS (log-rank *P* = 0.03159, Figure 7K). The distribution of risk score, the survival status, and the gene expression levels were similar to those in the training cohort (Supplementary Figures 5A–C). AUCs of the risk score model were 0.62, 0.607, 0.63, and 0.619 for 1-, 3-, and 5-year and median survival time, respectively. The C-index of the risk score model was 0.61 (95% CI: 0.52–0.71, *P* = 0.0229). These results implied that the risk score model was validated in the testing cohort and could be used to predict OS of patients with sarcoma (Supplementary Figure 5D).

We further validated the previously constructed nomogram in the testing cohort (Supplementary Figure 5E). AUCs were 0.791, 0.749, 0.738, and 0.761 for 1-, 3-, and 5-year and median survival time, respectively, and the C-index was 0.75 (95% CI: 0.68–0.81, *P* = 4.92e-14) (Supplementary Figure 5F). Furthermore, the time-dependent calibration plots showed a similar proximity between the bias-corrected lines and the standard line in the 1-, 3-, and 5-year time periods (Supplementary Figure 5H). The DCA analysis was further performed by using the total sarcoma cases for assessing clinical judgment utility of the risk score model and nomogram. As shown in Supplementary Figure 5I, the nomogram curve showed the highest net benefit. Taken together, the nomogram comprising the risk score model and clinical characteristics was an excellent model for predicting short-term or long-term OS in sarcoma patients, which might guide the therapeutic strategy decision in sarcoma patients’ treatment and long-term prognosis observation.

### Multidimensional Validation of the TCGA Dataset and External Databases

**FIGURE 8:**
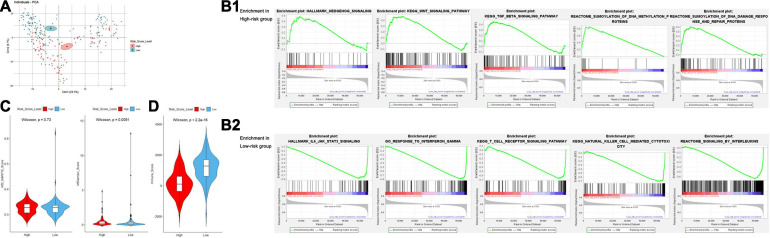
**(A)** Principal component analysis for examination of the gene expression diversity between the high- and low-risk groups. **(B)** Gene set enrichment analysis of the high- **(B1)** and low- **(B2)** risk groups. **(C)** Violin plots for the difference of MANTIS and MSIsensor scores between the high- and low-risk groups. **(D)** Violin plots for the difference of ESTIMATE-Immune scores between the high- and low-risk groups.

To further explore the significance of the risk score model, we performed multidimensional investigation using the TCGA dataset and external databases. Principal component analysis was performed using the log_2_(normalized counts data + 1) of the TCGA sarcoma cohorts. Furthermore, the result showed that there existed an obvious gene expression diversity between samples in the high- and low-risk groups (Figure 8A). We further explored the Oncomine database for the expression of genes in our risk score model (Supplementary Figures 6A,B). Compared to the non-tumor tissues, the expression levels of both IRF-1 and MFNG were significantly lower in 11 types of sarcoma including leiomyosarcoma, myxoid/round cell liposarcoma, and malignant fibrous histiocytoma. By accessing the online databases R2: Genomics Analysis and Visualization Platform, Serverless, and Logic, we found that both expression levels of IRF1 and MFNG were negatively associated with patients’ metastasis-free survival (MFS) and OS in various sarcomas (Supplementary Figures 6C–E, datasets: GSE42352, GSE21050, and GSE71118).

### Genetic Molecular Characteristics of the High- and Low-Risk Groups

Gene set enrichment analysis (GSEA) was performed to obtain a novel understanding on the diverse biological effects and specific pathways between high- and low-risk groups. We performed a standard GSEA using the normalized count data in several aspects, such as the hallmark gene sets, canonical pathways gene sets (KEGG and Reactome), and ontology gene sets (GO biological process). Samples in the high-risk group were enriched with various gene sets including DNA methylation, DNA damage response/repair, and oncogenesis-related pathways such as Wnt/β-catenin signaling, Hedgehog signaling, and TGF-β signaling pathway (Figure 8B1). However, the gene sets of the low-risk group were mainly enriched in immune-related pathways and biological processes such as interleukin production, regulation of immune response, NK cell-mediated cytotoxicity, interferon response, and TCR signaling (Figure 8B2). Detailed GSEA results are listed in Supplementary Table 7.

Microsatellite instability (MSI) is a biological characteristic indicating the genetic hypermutability of the genomic microsatellites, which is frequently studied in several types of cancer including sarcoma. To gain a further biological insight into genetic hypermutability, we used the computational scores *via* MANTIS and MSIsensor algorithms (Niu et al., 2014) and found no significant difference of MSI MANTIS scores between high- and low-risk groups. Although we observed a difference of MSIsensor scores between high- and low-risk groups (*P* = 0.0091), the overall MSI scores were relatively low (<3.5), indicating that sarcoma samples were mostly microsatellite stable (Figure 8C and Supplementary Table 7).

### Immune Microenvironment Analysis Between High- and Low-Risk Groups

Recently, the important role of exosome from tumor cells or immune-infiltrating cells in ceRNA networks has drawn arising interest in exploring TME regulatory mechanisms and developing promising therapeutic targets. Thus, we accessed the exoRBase database to show higher mRNA expression levels of IRF1 and MFNG in exosomes from human blood, compared to that from multiple tumor tissues (Supplementary Figure 6F).

**FIGURE 9:**
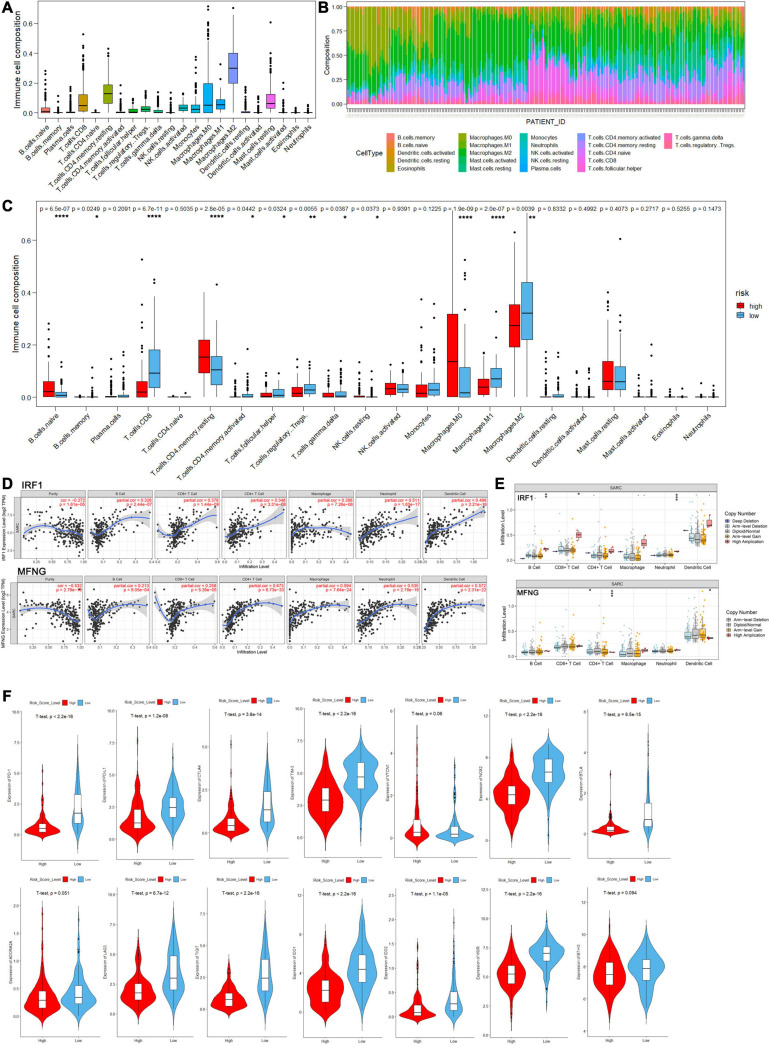
The landscape of immune cell infiltration in the high- and low-risk score groups of sarcoma patients by analysis of CIBERSORT. **(A)** Distribution proportion of each type of immune cell infiltration in all patients. **(B)** Relative proportion of immune cell infiltration in each sarcoma patient. **(C)** Bar plot visualizing significantly differentially infiltrated immune cells between high- and low-risk score group patients. **(D,E)** Correlation between immune cell infiltration and gene expression/mutation levels of IRF1 and MFNG analyzed by the TIMER database. **(F)** Violin plots of log-scaled normalized expression of inhibitory immune checkpoints between high and low risk groups.

In aspects of immune infiltrating intensity, the immune score calculated by ESTIMATE was significantly correlated with risk score (*P* < 2.2e-16) (Figure 8D). For the infiltrating abundances of various immune cell types, a bioinformatic tool, CIBERSORT, was used to identify several major types of immune cell infiltration in sarcoma samples to different degrees (Figures 9A,B and Supplementary Table 7). In the high-risk group with poor prognosis, naive B cells, resting memory CD4^+^ T cells, and non-activated macrophages (M0) were infiltrated with relatively higher levels. Functional cells in tumor immune response such as CD8^+^ T cells and pro-inflammatory macrophages (M1) were infiltrated with relatively higher levels in the low-risk group. However, regulatory T cells (Treg) and anti-inflammatory macrophages (M2) which may help tumor cells in immune evasion were also found at a relatively higher infiltrating level in the low-risk group (Figure 9C).

Moreover, exploring the relationship between immune infiltration and gene expression/mutation by TIMER database, we found that the expression levels of IRF1 and MFNG were positively correlated with the infiltration of B cells, CD8^+^ T cells, CD4^+^ T cells, and macrophages. Besides, IRF1 mutation was associated with the infiltration of B cells and CD4^+^ T cells; and MFNG mutation was associated with infiltration of CD8^+^ T cells and CD4^+^ T cells (Figures 9D,E).

Agents targeting immune checkpoints, such as PD-1 receptor and its ligand PD-L1, have transformed the treatment of many solid tumors by reversing immunosuppressive TME, but adoption in sarcoma has been in slow progress. Efforts are underway to determine which sarcoma patients will benefit from immune checkpoint blockade (ICB). Therefore, we investigated the expression of several inhibitory immune checkpoints between high- and low-risk sarcoma patients and found that the expression levels of most checkpoints such as PD-1, PD-L1, CTLA-4, TIM-3, LAG-3, and TIGIT in the high-risk group were significantly lower than in the low-risk group (*P* < 0.05), indicating higher immune cell infiltration in the TME of the low-risk group. However, the expression levels of specific inhibitory immune checkpoints such as VTCN1, B7-H3, and ADORA2A were close between high- and low-risk groups (Figure 9F).

## Discussion

Although most sarcoma harbors distinct biologic features, the primary treatment approach for locally advanced or unresectable disease often incorporates cytotoxic chemotherapy (Hashimoto et al., 2020; Heng et al., 2020; Lin et al., 2020). Recently, understanding of subtype-specific cancer biology has expanded and revealed distinct molecular alterations responsible for tumor initiation and progression (Grünewald et al., 2020; Steele and Pillay, 2020; Zhu et al., 2020), so has the study on cross talk between sarcoma cells and TME, as well as the heterogeneous mechanisms of tumor immune evasion (Becht et al., 2016; Pollack et al., 2018; Heymann et al., 2019; Miyake et al., 2020). These findings have motivated the development of targeted therapies in several ongoing subtype- or biomarker-specific clinical trials (Pollack et al., 2018; Dyson et al., 2019; Miwa et al., 2019; Heymann et al., 2020; Peyraud and Italiano, 2020). However, we still have not found validated biomarkers for predicting sarcoma patients’ response to immunotherapy and OS. Therefore, our study was conducted to identify prognostic biomarkers related to TME in sarcoma, so that we can harness subtype-specific insights into cancer and immune biology and bring more effective, less toxic therapeutic strategies to the clinic.

The cross talk between sarcoma cells and TME fuels the tumor progression, by inducing a local immunosuppressive environment and regulating proliferation, migration, drug resistance, dissemination, and/or dormancy of sarcoma cells (Heymann et al., 2019). In our study, we applied ESTIMATE to evaluate the tumor purity and immune/stromal cell infiltration in 259 sarcoma patients from the TCGA database, divided them into high- and low-immune score groups using median as the cutoff value, and identified DE-lncRNAs/miRNAs/mRNAs to construct a ceRNA network. Combining with the key immune infiltration-related gene modules by WGCNA, we constructed an immune infiltration-related ceRNA subnetwork (14 lncRNAs, 13 miRNAs, and 23 mRNAs), as well as a prognostic risk score model (IRF1, MFNG, hsa-miR-378a-5p, and hsa-miR-940).

The anti-tumorigenic role of interferon regulatory factor 1 (IRF1) has been reported in several types of cancer, by regulating genes related to PD-L1, DNA damage, apoptosis, and lymphocyte differentiation, also interacting multiple signaling pathways (Forero et al., 2019; Huang et al., 2019; Ohsugi et al., 2019; Wu et al., 2020). Moreover, IRF1 expression in tumor cells was also reported to be critical for the immune response to adoptive T cell therapy, as well as macrophage infiltration and memory CD4^+^ T cell activation (Wu et al., 2020). Zhang et al. found that Manic Fringe (MFNG) was highly expressed in Claudin-low breast cancer and functioned as an oncogene by activating Notch signaling, thereby promoting tumor cell migration, tumorsphere formation, and epithelial-to-mesenchymal transition (EMT)

(Zhang et al., 2015). Besides, MFNG was shown to be essential for optimal T and B cell development, such as promoting Th1 cell development and inhibiting Th2 cell development (Gu et al., 2012; Song et al., 2016). As for hsa-miR-378a-5p, it is reported that miR-378a can target SIRP alpha, thereby regulating the levels of inflammatory cytokines, as well as macrophage phagocytosis and polarization (Chen et al., 2019). Besides, miR-378a-5p was found to work as a tumor suppressor gene in renal cell carcinoma and colorectal cancer (Li et al., 2014; Liu et al., 2019). As for hsa-miR-940, it is reported that miR-940 can target MyD88 and inactivate MyD88/NF-κB signaling pathway, thereby regulating the inflammation through IL-1β induction (Cao et al., 2019). Meanwhile, miR-940 has also been demonstrated to be remarkably downregulated in hepatocarcinoma tissues and suppress tumor cell invasion and migration through regulating chemokine CXCR2 (Liu et al., 2018; Li et al., 2019).

Additionally, we demonstrated that risk score remained an independent prognostic factor after the modification of clinical characteristics, suggesting the promising potential of local immune status in accurate prognosis. Therefore, we combined risk score and other clinical features (age, tumor margin status, local recurrence, and metastasis at diagnosis), to develop a nomogram predicting OS probability of sarcoma patients in the 1-, 3-, and 5-year time periods. Based on the results of the calibration curves and DCA, our nomogram provides a complementary perspective on individualizing tumors, thus arising to be a promising tool for clinicians in the future.

With GSEA, we revealed that the high-risk group was enriched with various gene sets including DNA methylation, DNA damage repair, and oncogenesis-related pathways such as Wnt/β-catenin signaling, Hedgehog signaling, and TGF-β signaling pathway, while the low-risk group was mainly enriched in immune-related pathways and biological processes, such as interleukin production, regulation of immune response, interferon response, NK cell-mediated cytotoxicity, and TCR signaling. These results indicated that low-risk sarcoma patients possessed an elevated immune response state while the high-risk group presented enhanced activation of oncogenesis-related signaling pathways.

According to the cancer immunoediting hypothesis, less immunogenic cancer cells are selected (immune selection) and immunosuppressive networks are established (immune escape), thus evading antitumor immune responses and promoting tumor development in immune-competent hosts. Here, we applied CIBERSORT to analyze the infiltrating abundances of various immune cell types based on the TCGA sarcoma RNA-seq data. Consistent with our previous results, resting cells showed higher infiltration in the high-risk group while more active immune cells were abundant in the low-risk group. Although the prognostic role of CD8^+^ T cells is inconsistent due to high tumoral heterogeneity, macrophages have been shown to play a crucial role in tumor immunomodulation, correlating with survival of multiple sarcomas. Tumor-associated macrophages (TAMs) can mediate protumor or antitumor effects depending on M1/M2 polarization (Heymann et al., 2019; Miyake et al., 2020). Tregs and other immunosuppressive populations within the TME have been identified as the main cause of impaired response to immunotherapy. However, the controversial results of high Tregs and M2-TAM infiltration in the low-risk group need further study. To better estimate the response to immunotherapy, we investigated the expression of inhibitory immune checkpoints (PD-1, PD-L1, CTLA-4, TIM-3, BLTA, ADORA2A, LAG-3, TIGIT, IDO-1, IDO-2, NOX2, VSIR, B7-H3, and VTCN1) (Dancsok et al., 2019) between high- and low-risk sarcoma patients. Furthermore, the results indicated that poor prognosis of high-risk patients is partially due to the global low-level immune infiltration and latent function of the specific inhibitory immune checkpoints.

Since monotherapy with PD-1 or CTLA-4 inhibitors showed modest improvement in sarcoma patients’ survival, novel combinations with cytotoxic agents, anti-angiogenic agents, etc., are undergoing active investigation to induce consistent and durable responses (Pollack et al., 2018; Gamboa et al., 2020). Recent publications have highlighted that the important roles of alternative immune checkpoints such as pro-apoptotic TIM-3 or anti-proliferative LAG-3, in T-cell exhaustion, partially explained the resistance to monotherapy with PD-1 or CTLA-4 inhibitors (Dancsok et al., 2019). Also, the connection between angiogenesis and tumor immunity has aroused strong interest to the therapy for sarcoma combining an anti-VEGF agent and immunotherapy (Wilky et al., 2019). The VEGF pathway has been shown to inhibit T cell and dendritic cell development and promote suppressive immune cell populations such as Tregs and MDSCs, thus preventing tumor immune response. Moreover, normalizing the tumor vasculature helps to traffic tumor-specific T cells into the tumor bed.

Our research provides insights into the immune infiltration and inhibitory immune checkpoint expression in sarcoma. However, it is noteworthy that some limitations came out since the conclusion was drawn from data on retrospective studies, and prospective studies are warranted to further confirm our results. In addition, functional and mechanistic studies of the genes in risk score model should be conducted to support their clinical application.

## Conclusion

In summary, for the first time, we identified and validated a risk score model based on both ceRNA network and tumor immune microenvironment. Moreover, a nomogram comprising the risk score model can assist clinicians to select individualized therapeutic strategies for sarcoma patients. Notably, the immune infiltration-related risk score model provides an immunological perspective to elucidate the mechanisms on tumor progression and potential clues in developing the immunotherapy for patients with sarcoma.

## Materials and Methods

### Data Selection and Acquisition

The study reported herein fully satisfies the TCGA publication requirements^1^. Gene expression data and the corresponding clinical data for sarcoma samples (Project: TCGA-SARC) were acquired from the TCGA website^2^ through the TCGAbiolinks R package (Colaprico et al., 2016) in R software (version 4.0.2^3^) and Rstudio software (Version 1.3.1073^4^). Sarcoma samples of primary tumors with matched clinical data were included in the present study. Among them, available gene expression quantification data (RNA-seq) of 259 samples were downloaded through the Illumina HT-seq workflow including the count data and the normalized FPKM (Fragments Per Kilobase of transcript per Million mapped reads) data. Available miRNA isoform expression quantification data (miRNA-seq) of 257 samples were downloaded through the BCGSC miRNA profiling workflow including the count data and normalized RPM (reads per million mapped reads) data. The latest HomosapiensGRCh38 annotation file^5^ was used for gene symbol annotation. Besides, we obtained the MSI assessment data of the TCGA sarcoma cohorts from the cBioPortal platform (Cerami et al., 2012; Bonneville et al., 2017)^6^.

### Identification of DEGs and miRNAs

The ESTIMATE algorithm (Yoshihara et al., 2013) (Estimation of Stromal and Immune cells in Malignant Tumor tissues using Expression data), a bioinformatic tool for assessing tumor purity and the presence of infiltrating stromal/immune cells in tumor tissues, was used to calculate the corresponding infiltrating scores of the 259 sarcoma samples in the present study. Samples were divided to two groups according to the median value of immune infiltration-related risk scores. After filtering out low-abundance data, the edgeR R package (Robinson et al., 2010) was applied to normalize the expression count data and identify differentially expressed miRNAs (DEmiRs) and DEGs including mRNAs and lncRNAs. The differential expression was defined with a |log_2_ fold change (FC)| > 1 and a false discovery rate (FDR) value < 0.05.

### Weighted Gene Co-expression Network Analysis

Weighted gene co-expression network analysis (WGCNA) (Maertens et al., 2018) is a commonly used algorithm for analyzing high-throughput gene expression data with different characteristics. It has been most widely used in mining gene co-expression networks and intramodular hub genes based on pairwise correlations in genomic applications. In the present study, we applied the WGCNA R package (Langfelder and Horvath, 2008) as the data exploratory technique to analyze key gene clusters that were most relevant to immune infiltration-related risk scores in sarcoma samples.

### Construction and Analysis of ceRNA Network

We selected differentially expressed mRNAs, lncRNAs, and miRNAs to construct the ceRNA network. For prediction of the mRNA–miRNA interaction, data from three databases—miRDB (Chen and Wang, 2020), microT-CDS (Paraskevopoulou et al., 2013), and miRTarBase (Hsu et al., 2011)—was used. These databases recorded mRNA–miRNA interactions based on both bioinformatic algorithm and experimental verification. Only mRNA–miRNA interactions recognized by all the three databases were retained. For prediction of the lncRNA–miRNA interaction, experimental verified data from LncBase v2 (Paraskevopoulou et al., 2016) (Experimental Module) was used. Then, an lncRNA–miRNA–mRNA ceRNA network was constructed based on the recognized interactions. Based on the result of WGCNA, mRNAs, and lncRNAs in the “yellow-green” module were considered to be most relevant to immune infiltration-related risk scores. All mRNAs and lncRNAs in the “yellow-green” module were used to select a ceRNA subnetwork which was considered highly correlated with immune infiltration in sarcoma samples. The TBtools and Cytoscape software (Shannon et al., 2003) (version: 3.8.1) were used for network analysis and visualization.

### PPI Network Construction and Gene Functional Enrichment Analysis

Based on the result of WGCNA, we delimited genes of module membership larger than 0.65 as the hub genes, which were of relatively high correlation in the immune infiltration-related risk score-related gene cluster. The intersection of these hub genes and DEGs was used to construct a protein–protein interaction (PPI) network based on the utilization of the STRING database (Bader and Hogue, 2003). The minimum required interaction score was set to be 0.9 (highest confidence). The Cytoscape plugin MCODE was utilized to explore highly interconnected clusters in a network. Besides, the clusterProfiler R (Yu et al., 2012) package was used for gene functional enrichment analysis including both overrepresentation analysis and GSEA. Analysis of Gene Ontology (GO) (Carbon et al., 2019), Kyoto Encyclopedia of Genes and Genomes (KEGG) (Ogata et al., 1999) pathway, and Reactome (Fabregat et al., 2018) pathway was contained in the present study. An FDR value of 0.05 was considered the statistically cutoff value.

### Construction and Validation of the Immune Infiltration Related Prognostic Risk Model

lncRNAs/miRNAs/mRNAs in the immune infiltration-related ceRNA subnetwork and hub genes identified by WGCNA were selected for construction of the immune infiltration-related prognostic risk model. We firstly split the sarcoma patients to training (*n* = 129) and testing cohorts (*n* = 128) randomly by using the Caret R package (Kuhn, 2008). The training cohort was used for the construction of the prognostic risk model. The testing cohort was used for internal validation.

The Survival R package (Therneau, 1997) was utilized to analyze the correlation between the normalized expression of objective gene sets and sarcoma patients’ OS. The univariate Cox regression analysis was used to screen genes of which the expression was associated with OS. Lasso (least absolute shrinkage and selection operator) regression analysis was considered a method for variable selection and regularization in order to enhance the prediction accuracy and interpretability of the statistical model. By using the glmnet R package (Friedman et al., 2010), we utilized Lasso regression for selection of key genes screened in the univariate Cox regression analysis. In the Lasso analysis, we set the maximum number of passes over the data for all lambda values as default (10^5^). Then, the multivariate Cox regression was carried out and according to the method of Akaike information criterion (AIC) (Yamaoka et al., 1978), we selected the optimal gene sets to construct a risk score model. For each sample, the risk score equals the sum of the normalized expression of each gene multiplying the regression coefficient calculated from multivariate Cox regression analysis.

Sarcoma patients in the training cohort were divided to high- and low-risk groups according to the median risk score of the prognostic model. Then, K–M survival analysis was used to test whether risk score level was associated with prognosis. To evaluate the predictive accuracy of the risk score model, the prognostic risk score model was evaluated with time-dependent receiver operating characteristic (ROC) curve analysis in 1, 3, and 5 years and the median survival time of all samples by using the survivalROC R package. Besides, Harrell’s concordance index (C-index) was calculated by using the survcomp R package (Schröder et al., 2011).

To verify whether the risk score model was an independent prognostic factor, univariate and multivariate Cox proportional hazards regression analyses were performed using the risk score and clinical parameters including age, gender, race, tumor depth, tumor margin status, tumor total necrosis percent, local recurrence, and metastasis at diagnosis. Then, all independent prognostic factors were retained to construct a prognostic nomogram for assessment of 1-, 3-, and 5-year survival probability for sarcoma patients by using the rms (Harrell, 2015) and mstate (de Wreede et al., 2011) R packages. The discriminative efficacy of the nomogram was evaluated by analyses of the time-dependent ROC curve (Heagerty et al., 2000) and C-index. The consistency of the nomogram was tested by time-dependent calibration plots. Furthermore, the clinical judgment utility of the risk score model and nomogram was evaluated *via* decision curve analysis (Vickers and Elkin, 2006) by using the rmda R package (Kerr et al., 2016).

As for the internal validation, all the above methods were used to evaluate the risk score model and nomogram in the testing cohort. The principal component analysis for sarcoma samples of high- and low-risk score groups was performed and visualized by using the psych and factoextra R packages (Revelle, 2017). Multidimensional external validation of the mRNAs and miRNAs composing the risk model was performed based on the online platforms including Oncomine (Rhodes et al., 2004), cBioPortal (Cerami et al., 2012), TIMER (Li et al., 2020), exoRBase (Li et al., 2018), SurvExpress (Aguirre-Gamboa et al., 2013), LOGpc^7^, and R2: Genomics Analysis and Visualization Platform^8^.

### Gene Set Enrichment Analysis

To investigate the enriched biological processes and signaling pathways that differ between sarcoma samples of the high- and low-risk score groups, the standard GSEA^9^ was performed by using the EdgeR-processed normalized count data. The annotated hallmark gene sets, canonical pathway gene sets (KEGG and Reactome) (Ogata et al., 1999; Fabregat et al., 2018), and Ontology gene sets (GO biological process) (Carbon et al., 2019) were selected as the reference gene sets. The threshold for GSEA was set at the nominal *P*-value < 0.05, FDR < 0.25, and | normalized enrichment score (NES) | >1.0. A significant positive NES presents that the gene set is mostly at the top of the ranked list of genes, which indicates the enrichment in the high-risk score group. A significant positive NES indicates the opposite.

### Data Analysis

All statistical data was analyzed in the R software (version 4.0.2). An independent *t*-test was applied for the comparison of log-transformed normalized expression data between two groups. Immune cell infiltration scores calculated *via* ESTIMATE and Cibersort and MSI scores obtained from cBioPortal were compared by the Wilcoxon test between two groups. Statistical tests were two-tailed with a statistical significance level set at *P* < 0.05. The ggplot2 R package (Wickham, 2016) was used for visualization.

## Data Availability Statement

The datasets presented in this study can be found in online repositories. The names of the repository/repositories and accession number(s) can be found in the article/Supplementary Material.

## Author Contributions

DS, ZZ, and ZS conceived and designed the study. DS and SM did the literature research, performed the study selection, data extraction, and statistical analysis, and wrote the draft. FP, BH, BZ, JL, and TH participated in the extraction and analysis of data. All the authors read and approved the final version of the manuscript.

## Conflict of Interest

The authors declare that the research was conducted in the absence of any commercial or financial relationships that could be construed as a potential conflict of interest.

## Footnotes

1 https://www.cancer.gov/tcga

2 https://portal.gdc.cancer.gov/repository

3 https://www.r-project.org

4 https://rstudio.com

5 https://www.ensembl.org

6 https://www.cbioportal.org/

7 http://bioinfo.henu.edu.cn/DatabaseList.jsp

8 http://r2.amc.nl

9 http://software.broadinstitute.org/gsea

## Supplementary Material

Supplementary Figure 1 Correlation between immune score and clinical characteristics in TCGA sarcoma patients: **(A)** tumor depth, **(B)** local disease recurrence, **(C)** tumor margin status, **(D)** radiation therapy, **(E)** metastasis at diagnosis, **(F)** tumor total necrosis percent.

Supplementary Figure 2 Heatmap and volcano plot of differentially expressed genes between the high and low immune score groups of the whole annotated RNA-Seq data.

Supplementary Figure 3 **(A)** Clustering for the detection of outlier samples in WGCNA. **(B)** Gene dendrogram and plot for the dynamic tree cutting. **(C)** Clustering of module eigengenes. **(D)** Eigengene expression in the yellow-green module for each sample. **(E)** The topological overlap measure (TOM) for gene network connections (left: all genes, right: randomly selected 1000 genes).

Supplementary Figure 4 The total PPI network visualized via Cytoscape.

Supplementary Figure 5 **(A–C)** Risk score curve, survival status and the gene expression levels for each patient were discretely distributed between two groups in the testing cohort. **(D)** Time-dependent ROC curves for the risk score model for predicting the survival probability of 1-, 3-, 5-year and median-survival time overall survival in the testing cohort. **(E)** Nomogram for predicting the survival probability of 1-, 3-, and 5-year overall survival in the testing group. **(F)** time-dependent ROC curves for the Nomogram in the testing group. **(G)** time-dependent ROC curves for the Nomogram in the training group. **(H)** The time-dependent calibration plots for the nomogram in 1,3,5-year time periods in the testing group. **(I)** The plot of DCA analysis for assessing clinical judgment utility of the risk score model and nomogram in the total TCGA sarcoma cohort.

Supplementary Figure 6 Multidimensional validation of the risk score model in external databases. **(A,B)** Expression of Both IRF1 **(A1–A7)** and MFNG **(B1–B13)** were downregulated in sarcomas compared to non-tumor tissues in Oncomine database. **(C)** R2: Genomics Analysis and Visualization Platform: both expression levels of IRF1 and MFNG were negatively associated with patients’ metastasis-free survival and overall survival in osteosarcomas (datasets: GSE42352). **(D)** SurvExpress: both expression levels of IRF1 and MFNG were negatively associated with patients’ metastasis-free survival in various sarcomas (datasets: GSE21050). **(E)** LOGpc: expression level of MFNG was negatively associated with patients’ metastasis-free survival in various sarcomas (datasets: GSE71118). **(F)** the mRNA expression levels of IRF1 and MFNG in exosomes were validated in human blood exosomes compared to multiple tumor tissues by using the exoRBase database.
