# Supplementary figures and images for: Development of a Novel Immune Infiltration-Related ceRNA Network and Prognostic Model for Sarcoma
Source: Front Cell Dev Biol. 2021 Jul 1;9:652300. doi: 10.3389/fcell.2021.652300 (PMC8281254; doi:10.3389/fcell.2021.652300)

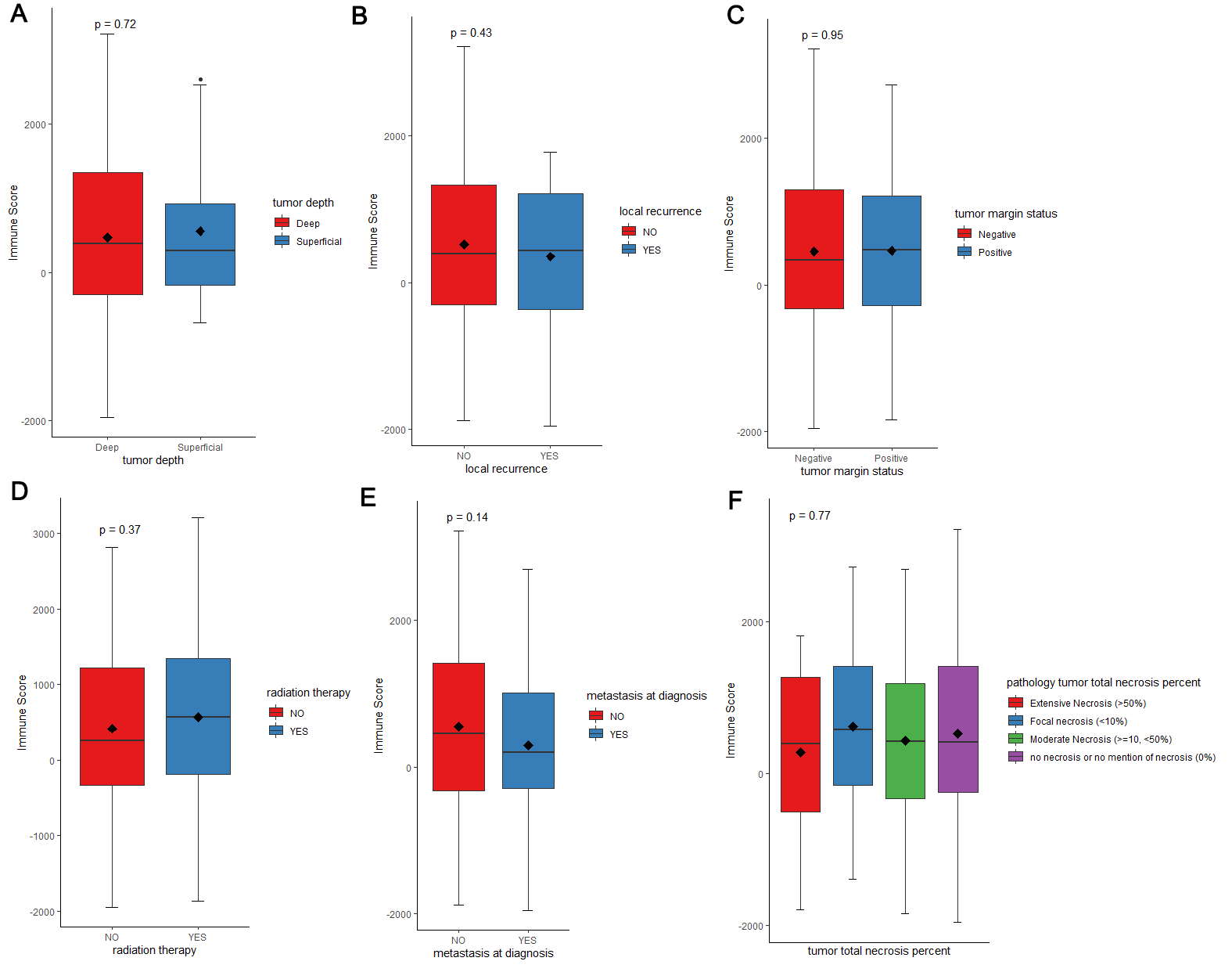

Supplement: Supplementary Figure 1 — Correlation between immune score and clinical characteristics in TCGA sarcoma patients: (A) tumor depth, (B) local disease recurrence, (C) tumor margin status, (D) radiation therapy, (E) metastasis at diagnosis, (F) tumor total necrosis percent. [file Image_1.TIF]

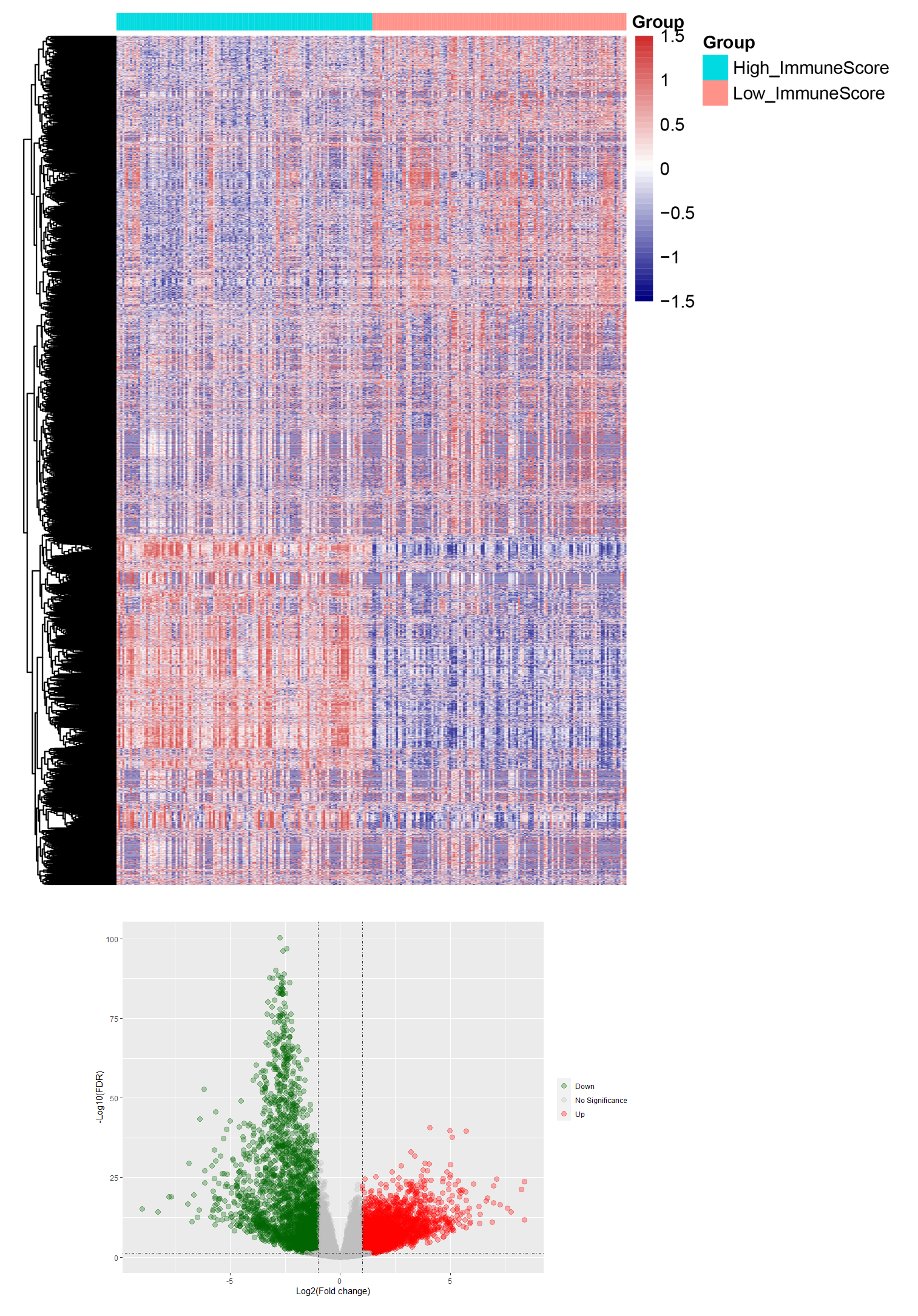

Supplement: Supplementary Figure 2 — Heatmap and volcano plot of differentially expressed genes between the high and low immune score groups of the whole annotated RNA-Seq data. [file Image_2.TIF]

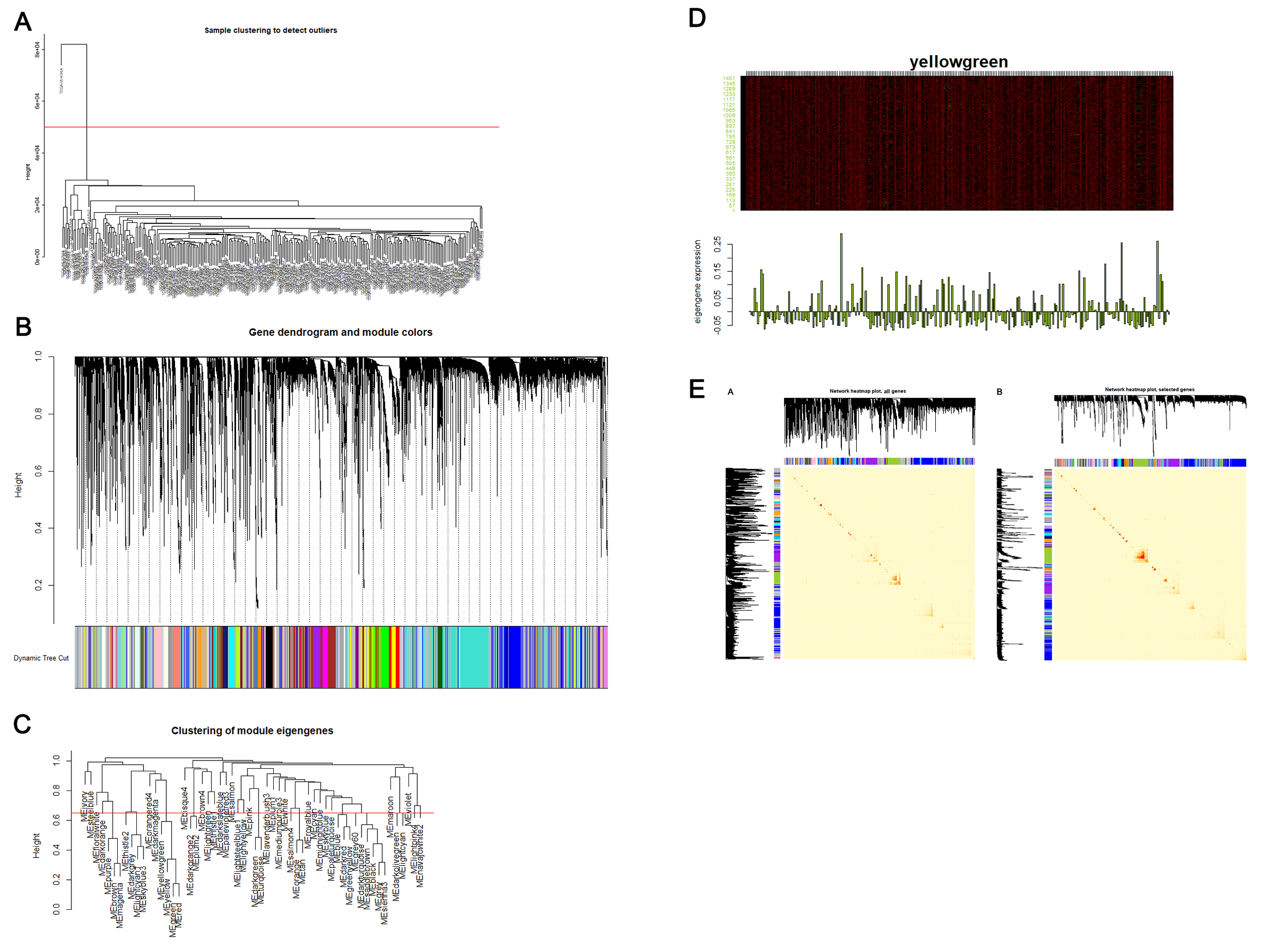

Supplement: Supplementary Figure 3 — (A) Clustering for the detection of outlier samples in WGCNA. (B) Gene dendrogram and plot for the dynamic tree cutting. (C) Clustering of module eigengenes. (D) Eigengene expression in the yellow-green module for each sample. (E) The topological overlap measure (TOM) for gene network connections (left: all genes, right: randomly selected 1000 genes). [file Image_3.TIF]

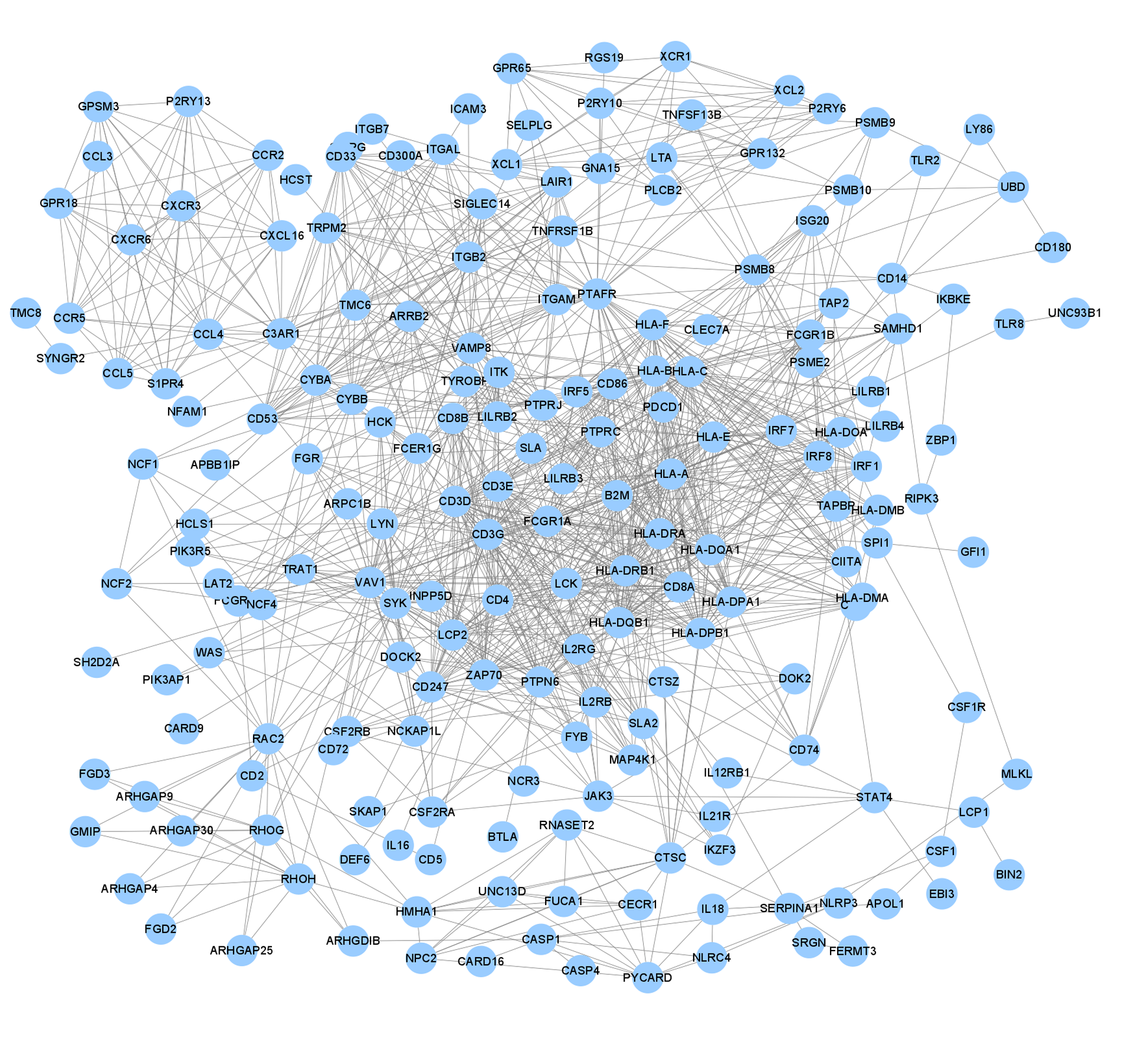

Supplement: Supplementary Figure 4 — The total PPI network visualized via Cytoscape. [file Image_4.TIF]

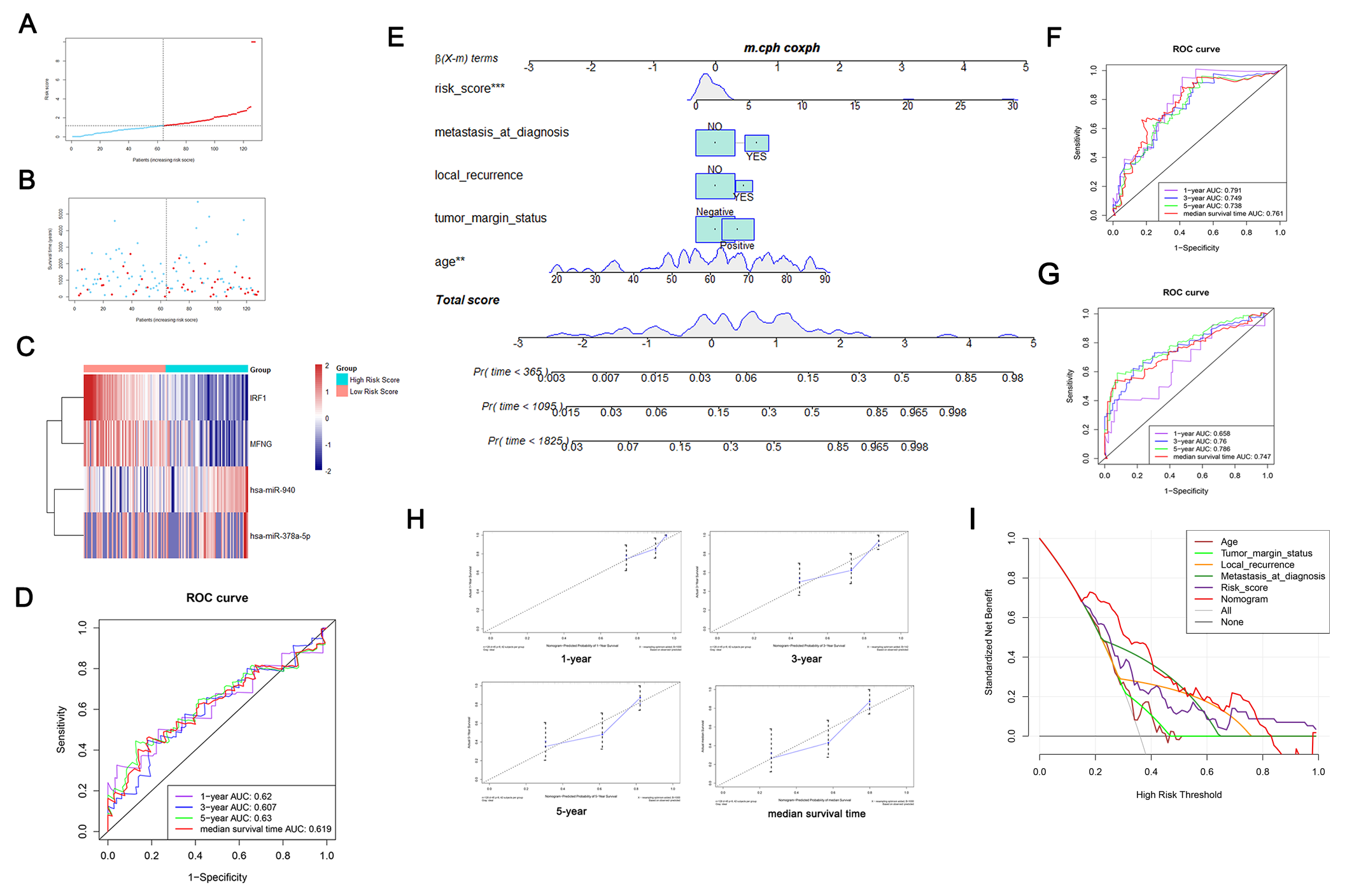

Supplement: Supplementary Figure 5 — (A–C) Risk score curve, survival status and the gene expression levels for each patient were discretely distributed between two groups in the testing cohort. (D) Time-dependent ROC curves for the risk score model for predicting the survival probability of 1-, 3-, 5-year and median-survival time overall survival in the testing cohort. (E) Nomogram for predicting the survival probability of 1-, 3-, and 5-year overall survival in the testing group. (F) time-dependent ROC curves for the Nomogram in the testing group. (G) time-dependent ROC curves for the Nomogram in the training group. (H) The time-dependent calibration plots for the nomogram in 1,3,5-year time periods in the testing group. (I) The plot of DCA analysis for assessing clinical judgment utility of the risk score model and nomogram in the total TCGA sarcoma cohort. [file Image_5.TIF]

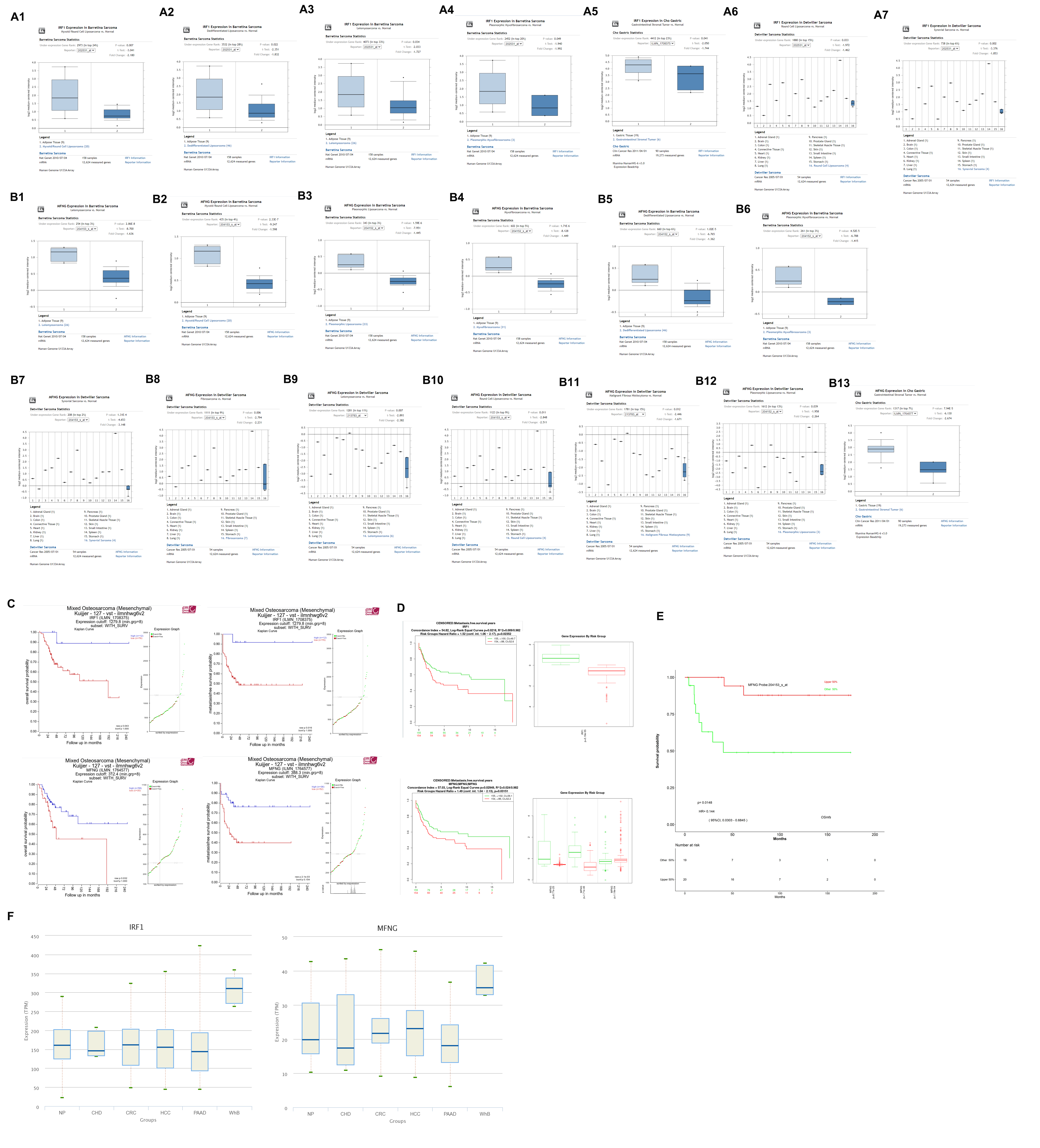

Supplement: Supplementary Figure 6 — Multidimensional validation of the risk score model in external databases. (A,B) Expression of Both IRF1 (A1–A7) and MFNG (B1–B13) were downregulated in sarcomas compared to non-tumor tissues in Oncomine database. (C) R2: Genomics Analysis and Visualization Platform: both expression levels of IRF1 and MFNG were negatively associated with patients’ metastasis-free survival and overall survival in osteosarcomas (datasets: GSE42352). (D) SurvExpress: both expression levels of IRF1 and MFNG were negatively associated with patients’ metastasis-free survival in various sarcomas (datasets: GSE21050). (E) LOGpc: expression level of MFNG was negatively associated with patients’ metastasis-free survival in various sarcomas (datasets: GSE71118). (F) the mRNA expression levels of IRF1 and MFNG in exosomes were validated in human blood exosomes compared to multiple tumor tissues by using the exoRBase database. [file Image_6.tif]
